# Perspectives on the economic costs of disease outbreaks to health systems

**DOI:** 10.11604/pamj.2026.54.4.52132

**Published:** 2026-05-07

**Authors:** George Nyadimo Agot, Marshal Mutinda Mweu

**Affiliations:** 1 Department of Public and Global Health, Faculty of Health Sciences, University of Nairobi, Nairobi, Kenya

**Keywords:** Perspectives, economic costs, disease outbreaks, zoonotic diseases, health systems

## Abstract

Disease outbreaks are non-random public health phenomena attributed to known or idiopathic human, animal, and animal-based factors; a realm referred to as the One Health concept; a new global public health order for control and prevention of epidemics, endemics, and pandemics. These are diseases of viral, prion, bacterial, fungal, and parasitic origin, transmitted between vertebrate animals and humans and vice versa. Emergence and re-emergence of zoonotic diseases such as rabies, Ebola, COVID-19, monkeypox, avian influenza, anthrax, brucellosis, leptospirosis, and Lyme disease continue to affect populations across the world, pointing to unexpected budget impacts of the affected countries.

## Opinion

Disease outbreaks undoubtedly exert an enormous economic burden on the fiscal national resource envelope, attributed to substantial economic costs of outbreak surveillance and related public health interventions [1-4]. Even though some of the infectious diseases have been eradicated or nearly wiped out from nature due to strategic medical and public health interventions, persistence, emergence, and re-emergence of new and old infectious diseases underscore the estimation of the economic costs of disease outbreaks to health systems [2]. Strategies aimed at controlling and preventing outbreaks of zoonotic diseases comprise public health, medical, and behaviour change interventions [1,2,5]. Cost drivers of medical interventions consist of diagnosis, treatment regimens, and personnel due to surging human capital and productivity loss [1,2,5]. Additionally, the cost drivers of public health interventions comprise surveillance, individual and environmental hygiene, human and animal vaccinations, whilst behaviour change interventions include mass media campaigns, Information, Education and Communication (IEC) materials [1,2,5].

The cost drivers, consisting of direct and overhead (shared) costs of the aforementioned interventions, are often gathered through the ingredient or activity-based costing (ABC) approaches from the health providers' economic perspective and categorized as recurrent and capital costs [1]. The process of gathering the economic costs of the above-named interventions constitutes identification, measurement, valuation, and comparison of costs of alternatives being considered, including the status quo [1]. Recurrent (variable) costs comprise the cost of routine personnel, personnel capacity surge, personnel productivity loss, as well as pharmaceutical, non-pharmaceutical, medical, non-medical, surgical, and non-surgical supplies, in addition to overhead costs [1]. Capital (fixed) costs, on the other hand, constitute diagnostic and imaging equipment, machinery, health and medical care infrastructure such as buildings, ambulances, and internet, among others [1]. Further, capital costs are considered for differential-time discounting, whilst statistical and/or sensitivity analysis is conducted to ascertain the robustness of the costing exercise findings because of possible uncertainties arising from sample size determination and data collection methods [1,6-9].

Finally, perspectives of economic costs of disease outbreaks to health systems should always be reported as per the CHEERS (Checklist for Consolidated Health Economic Evaluation Reporting Standards) guidelines [10]. Estimating economic costs of zoonotic disease outbreaks from a health systems perspective is of great global significance in understanding their impact on public health resource envelope, as well as informing and developing public health policies with a view to formulating system-specific mitigation preparedness [1,2].
